# PLPPR4 haploinsufficiency causes neurodevelopmental disorders by disrupting synaptic plasticity via mTOR signalling

**DOI:** 10.1111/jcmm.17899

**Published:** 2023-08-07

**Authors:** Huanzheng Li, Qian Zhang, Ru Wan, Lili Zhou, Xueqin Xu, Chenyang Xu, Yuan Yu, Yunzhi Xu, Yanbao Xiang, Shaohua Tang

**Affiliations:** ^1^ Human Aging Research Institute Nanchang University Nanchang China; ^2^ Wenzhou Central Hospital Wenzhou China; ^3^ Pediatric Genetics Zhejiang Provincial People's Hospital Hangzhou China

**Keywords:** intellectual disability, iPSCs, mTOR, neurodevelopmental disorder, *PLPPR4*

## Abstract

Phospholipid phosphatase related 4 (PLPPR4), a neuron‐specific membrane protein located at the postsynaptic density of glutamatergic synapses, is a putative regulator of neuronal plasticity. However, PLPPR4 dysfunction has not been linked to genetic disorders. In this study, we report three unrelated patients with intellectual disability (ID) or autism spectrum disorder (ASD) who harbour a de novo heterozygous copy number loss of *PLPPR4* in 1p21.2p21.3, a heterozygous nonsense mutation in *PLPPR4* (NM_014839, c.4C > T, p.Gln2*) and a homozygous splice mutation in *PLPPR4* (NM_014839: c.408 + 2 T > C), respectively. Bionano single‐molecule optical mapping confirmed *PLPPR4* deletion contains no additional pathogenic genes. Our results suggested that the loss of function of PLPPR4 is associated with neurodevelopmental disorders. To test the pathogenesis of *PLPPR4,* peripheral blood mononuclear cells obtained from the patient with heterozygous deletion of *PLPPR4* were induced to specific iPSCs (CHWi001‐A) and then differentiated into neurons. The neurons carrying the deletion of *PLPPR4* displayed the reduced density of dendritic protrusions, shorter neurites and reduced axon length, suggesting the causal role of PLPPR4 in neurodevelopmental disorders. As the mTOR signalling pathway was essential for regulating the axon maturation and function, we found that mTOR signalling was inhibited with a higher level of p‐AKT, p‐mTOR and p‐ERK1/2, decreased p‐PI3K in PLPPR4‐iPSCs neurons. Additionally, we found silencing *PLPPR4* disturbed the mTOR signalling pathway. Our results suggested PLPPR4 modulates neurodevelopment by affecting the plasticity of neurons via the mTOR signalling pathway.

## 1 INTRODUCTION

Intellectual disability (ID) is frequently associated with comorbidity in autism spectrum disorders (ASD) and is often characterized by limitations in intellectual functioning and adaptive behaviour.^1^ ID and ASD affect 3%–5% of the global population.^2^ Genetic causes account for at least 30% of ID and ASD cases, and the mechanism is largely unknown. Genomic technologies have significantly accelerated the discovery the genetic aetiologies of these diseases; however, many ID and ASD‐associated genes remain unknown. Many ID and ASD genes share common pathways, and ID and autism‐associated genes are related to nervous system development.^3^ Previously, we had described a patient diagnosed with mild ID and dysmorphic facial features who harboured a potentially pathogenic phospholipid phosphatase related 4 (*PLPPR4*) deletion with de novo inheritance.^4^

PLPPR4, a lipid phosphate phosphatase (LPPs) superfamily member, is specifically expressed in neurons and is located in the membranes of outgrowing axons. It plays a specific role in excitatory synapse terminating on glutamatergic neurons.^5^, ^6^ PLPPR4 interacts with bioactive phospholipids, such as LPA, which can signal to presynaptic LPA_2_–receptors.^7^ PLPPR4 can increase the dephosphorylation of exogenous LPA, attenuate phospholipid‐induced axon collapse in neurons, and facilitate outgrowth in the hippocampus.^8^ The deletion of PLPPR4 results in severe hippocampal overexcitability, leading to epileptic discharge in juvenile animals^7^ *PLPPR4* knockout mice are viable but show transiently reduced brain weight during development, increased grooming activity and decreased learning activity compared to their wildtype littermates.^7^, ^9^ Thus, PLPPR4 has been suggested to be potentially involved in neurodevelopmental processes, exhibiting ID and/or autism phenotypes.

The mTOR signalling cascades were initially identified as cancer regulatory pathways but have now been demonstrated to be critical for synaptic plasticity and behaviour.^10^ mTOR is a protein kinase involved in translation control and long‐lasting synaptic plasticity. Activation of mTOR has been functionally linked with local protein synthesis in synapses, resulting in the production of proteins required to form, mature and function new spine synapses.^11^ In addition, PLPPR4/LPA regulates synaptic plasticity, and it has been shown that LPA regulates processes such as the activation of ERK, mTOR, cell division, Ca^2+^ transients, etc.^12^ Dysregulation of signalling in either direction can be detrimental, indicating that the balance between signalling pathways is vital for maintaining homeostasis synaptic plasticity. Therefore, exploring the effect of PLPPR4 on neuroplasticity via the mTOR signalling pathway is necessary.

Here, we report two additional patients with mutations in the *PLPPR4* gene. The mechanisms that the mutations in the *PLPPR4* gene cause neurodevelopmental disorders were tested in neurons differentiated from patients' iPSCs. PLPPR4 functions were examined in dendritic spine formation in iPSCs‐neurons. Furthermore, the regulatory role of PLPPR4 in the mTOR signalling pathway was also examined.

## 2 METHODS

### 2.1 Ethics statement

We investigated a patient from Jiangxi Province, China, diagnosed with mild ID (IQ = 46 on the China‐Wechsler Intelligence Scale) and dysmorphic facial features, hypertelorism, and strabismus. The patient carried a deletion at 1p21.2p21.3, containing only one coding gene *PLPPR4*.^4^ Clinical evaluations and peripheral blood mononuclear cells (PBMCs) were collected from the family members after obtaining informed written consent approved by the Ethics Committee of the Wenzhou Central Hospital, China (L2022‐04‐097).

### 2.2 Single‐molecule optical mapping

The karyotype of this patient is 46, XX, t(1;8;10)(p21;p21;p13). Array analysis showed a 0.21‐Mb deletion at 1p21.2p21.3. We applied SMOM technology to detect gene fracture sites to confirm no other functional gene was present. High‐molecular‐weight (HMW) DNA was extracted from 2 mL of fresh (<5 days) peripheral blood following the manufacturer's instructions (IrysPrep Experienced User Card Human Blood Protocol, Bionano Genomics, USA). DNA was quantified using the Qubit dsDNA BR assay with the Qubit 3.0 fluorometer (Thermo Fisher Scientific). DNA labelling was performed using the Bionano Prep Direct Label and Stain (DLS) kit (Bionano Genomics) following the manufacturer's instructions. In brief, 750 ng of megabase‐size gDNA was labelled with Direct Label Enzyme‐1 (Bionano Genomics), which does not introduce nicks. About 80 ng HMW DNA was loaded onto a Saphyr chip and tested on the Saphyr system (Bionano Genomics) for 24 h. Thousands of high‐resolution images of fluorescently labelled individual DNA molecules were acquired by the Saphyr detectors. After collecting the data, Bionano Solve/Access software (Bionano Genomics) was used to align the labelled molecules against the labelled reference sequence (hg19) and to identify the structural variation signatures.

### 2.3 Establishment and identification of ID‐iPSCs with *PLPPR4* heterozygous deletion

The iPSC line was established from the PBMCs of patient 1, and cells were infected with retroviral vectors containing Oct4, Sox2, Klf4 and c‐Myc. iPSCs were generated as described previously.^13^ The pluripotency of the generated iPSC lines was assessed by immunostaining of pluripotency markers (Nanog, Oct4, TRA‐1‐60 and Sox2). Cells were cultured at 37°C under an atmosphere of 5% CO_2_.

### 2.4 Differentiation of iPSCs to neurons

The iPSCs were passaged and cultured on Matrigel‐coated dishes with mTeSR Medium until they reached the desired number of cells. EDTA was used to separate the cells in this stage. iPSCs culture and differentiation media were used according to different stages of iPSCs differentiation. Cells were cultured at 37°C under an atmosphere of 5% CO_2_. Fresh Medium was replaced every 2 or 3 days. Map2, NeuN, Tuj1 and PLPPR4 were all detected at 28 days after differentiation. Control and experimental samples were collected from differentiations performed in parallel with iPSCs obtained from the same maintenance batch.^14^

### 2.5 Immunofluorescence staining

The expression of Map2 (Sigma, M4403, 1:2000), NeuN (Abcam, ab177487, 1:10,000), Tuj1 (Biolegend, 845502, 1:2000), Tau (Abcam, ab32057, 1:1000) and PLPPR4 (Bioss, bs‐11875R, 1:1000) was detected in differentiated neurons. Cells were fixed with 4% (w/v) paraformaldehyde for 30 min at room temperature and washed with PBS three times (5 min each). After permeabilizing with 0.5% (v/v) Triton X‐100 for 20 min, the cells were washed with PBS three times (3 min each). Samples were incubated with 5% (v/v) goat serum (Solarbio) for 1 h at room temperature and incubated with primary antibodies overnight at 4°C. The cells were incubated with secondary antibodies for 1 h at room temperature the following day and observed with a BX51 fluorescent microscope (Olympus). Quantification of the immunofluorescent signals was performed with ImageJ software. Axon length was quantified using Fiji software,^15^ and the axon could be defined reliably as the longest neurite, which was at least three times longer than the second longest neurite. Axon length was determined by measuring the length of the primary axon (the longest axon) from the cell body to the centre of the axon growth cone, not including the spindle branch. The number of dendrites was counted manually.^16^

### 2.6 Cell culture and transfection

The human neuroblastoma cell line (SH‐SY5Y) was cultured in DMEM containing 10% foetal bovine serum, 100 IU/mL penicillin and 100 μg/mL streptomycin. SH‐SY5Y were grown in 6‐well plates and were incubated in a saturated humidity incubator at 37°C with 5% CO_2_. According to the instructions, the PLPPR4 shRNA plasmid was transfected into SH‐SY5Y cells using Lipofectamine 3000.

### 2.7 Western blotting

Cells were lysed in ice‐cold RIPA buffer (Beyotime Biotechnology) and supplemented with phenylmethanesulfonyl fluoride (Beyotime Biotechnology). Protein concentrations were determined using the BCA protein assay kit (Beyotime Biotechnology). Total proteins were resolved by sodium dodecylsulfate–polyacrylamide gel electrophoresis and transferred to PVDF membranes (Millipore). After blocking, the membranes were incubated with a primary antibody and detected with a horseradish peroxidase‐conjugated secondary antibody. Proteins were visualized using ECL Western blotting detection kit (Advansta). Immunoreactive bands were quantified using Image Quant TL software. The following antibodies were used: anti‐PLPPR4 (Jackson, sc‐377263, 1:1000), anti‐phospho‐p44/42 MAPK (ERK1/2) (Cell Signaling Technology, 4370 s, 1:1000), anti‐p44/42 MAPK (ERK1/2) (Cell Signaling Technology, 4659, 1:1000), anti‐phospho‐mTOR (Cell Signaling Technology, 2971S, 1:1000), anti‐mTOR (Cell Signaling Technology, 2983S, 1:1000) and anti‐GAPDH (Cell Signaling Technology, 5174S, 1:1000). All antibodies were diluted in PBS containing 5% skimmed milk and 0.1% Tween‐20.

### 2.8 Statistical analysis

The significance of the differences between groups was evaluated using unpaired two‐tailed Student's *t*‐test or one‐way anova with Tukey post hoc test. *p*‐Values < 0.05 were considered statistically significant. Data are presented as mean ± standard error of the mean (SEM), and the number of experiments performed with independent cultures or animals (*n*) and *p*‐values are indicated in the figure legends. Box and whisker plots show the mean (+), median (horizontal line in box) and maximum values.

## 3 RESULTS

### 3.1 Clinical presentation

**FIGURE 1:**
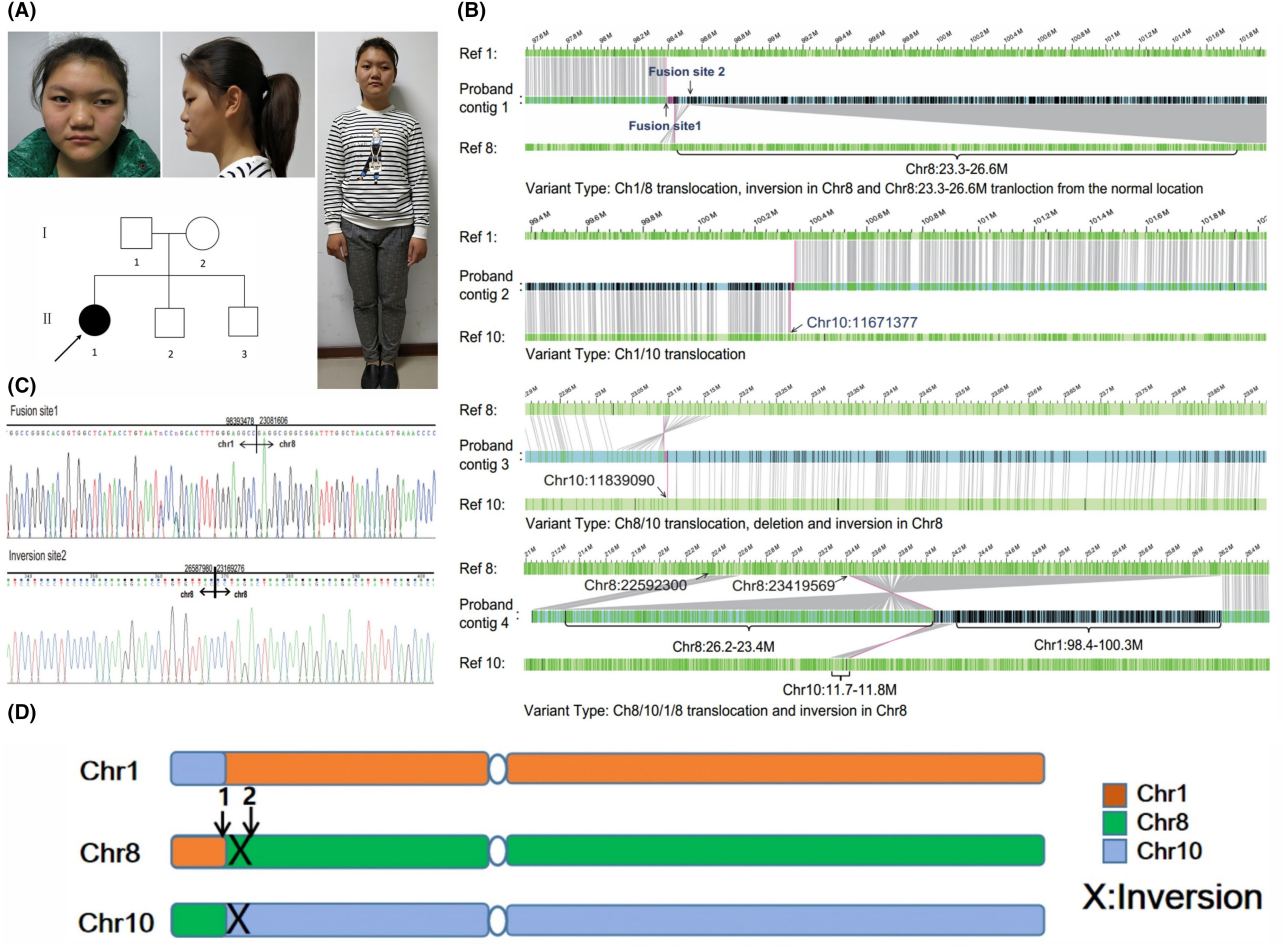
Clinical features and pathogenic analysis of Patient #1 (A) Photographs and pedigree of the ID patient. Filled symbols indicate the affected individuals (arrow). (B) The green bar represents the reference chromosome, and the blue bar represents the sample map generated based on long molecule assembly of the proband's genome. Vertical lines indicate direct label enzyme‐1 labelled sites and corresponding matches between reference (green) and sample (blue) genomes. Lines between reference and assembled maps show the alignment of the two maps. The structural variation type is shown below each map. (C) Fusion site 1 and inversion site 2 were verified by Sanger sequencing. (D) Schematic diagram of the Bionano Prep Direct Label and Stain procedure.

The clinical phenotypes of the three patients are summarized in Table 1. All patients showed neurodevelopmental disorders, mild ID and ASD. Patient 1 is a 15‐year‐old girl with mild ID (IQ = 46 on the China‐Wechsler Intelligence Scale) and dysmorphic facial features, including hypertelorism and strabismus (Figure 1A), who attended Wenzhou Central Hospital for genetic counselling. She was born prematurely and weighed less than 2 kg. The score of Gesell Developmental Schedules is 59. She has delayed language and motor development, poor expression ability, uncoordinated walking, difficulty walking in a straight line, inability to walk with small steps, and prone to falling when turning and backward. When younger, she exhibited aggressive behaviours (e.g. hair grabbing and needle pricking), which decreased in frequency as she aged. She has such poor mathematical skills that she cannot add or subtract. She has left exotropia, and both eyes' convergence is poor. Her optic nerve is atrophied, and her sensitivity to the visual field in her left eye is irregularly reduced. The heel–knee‐tibia test of both lower limbs is clumsy. Video electroencephalogram (VEEG) data showed a slowing down of alpha rhythm. Her karyotype is 46, XX, t (1;8;10) (p21;p21;p13). Array analysis showed a 0.21‐Mb deletion at 1p21.2p21.3, including deletion of the coding gene *PLPPR4* (Table 1).^4^

**TABLE 1:** Clinical and genetic findings in three patients carrying *PLPPR4* variants.

Patient	Patient no. 1	Patient no. 2	Patient no. 3
Genetic variation	1p21.2p21.3(99580912–99794788) × 1, *PLPPR4* deletion, de novo	*PLPPR4* (NM_014839, c.4C > T, p.Gln2*), de novo	*PLPPR4* (NM_014839: c.408 + 2 T > C), unknown
Age at diagnosis	11 years	2 years	8 months
Patient gender	Female	Male	Male
Height	Normal	Short status	Short status
DD/ID	Mild ID	Mild ID	Mild ID
Language	Delay	Disorder	Delay
Social communication	Poor	Poor	Poor
Walking ability	Uncoordinated	−	−
Motor development	Delay	Delay	Delay
Autistic behaviours	+	+	+
Aggressive behaviours	+	−	−
Mathematical skills	Poor	Poor	Poor
Motor skills	Delay	Delay	Delay
Ophthalmic Diseases	Left exotropia and poor convergence	Normal	Cortical visual impairment
Optic nerve	Atrophied	−	+
Visual field	Reduced in the left eye	−	+
VEEG	Slowing down of alpha rhythm	Normal	Infantile spasms, hypsarrhythmia
Hypomyotonia	+	+	+
Stutter	−	+	−
Seizure	−	+	+
Dysmorphic facial	+	−	+
Macrocephaly	−	−	+

Abbreviations: ASD, autism spectrum disorder; DD, Developmental delay; ID, intellectual disability; VEEG, video electroencephalogram.

**FIGURE 2:**
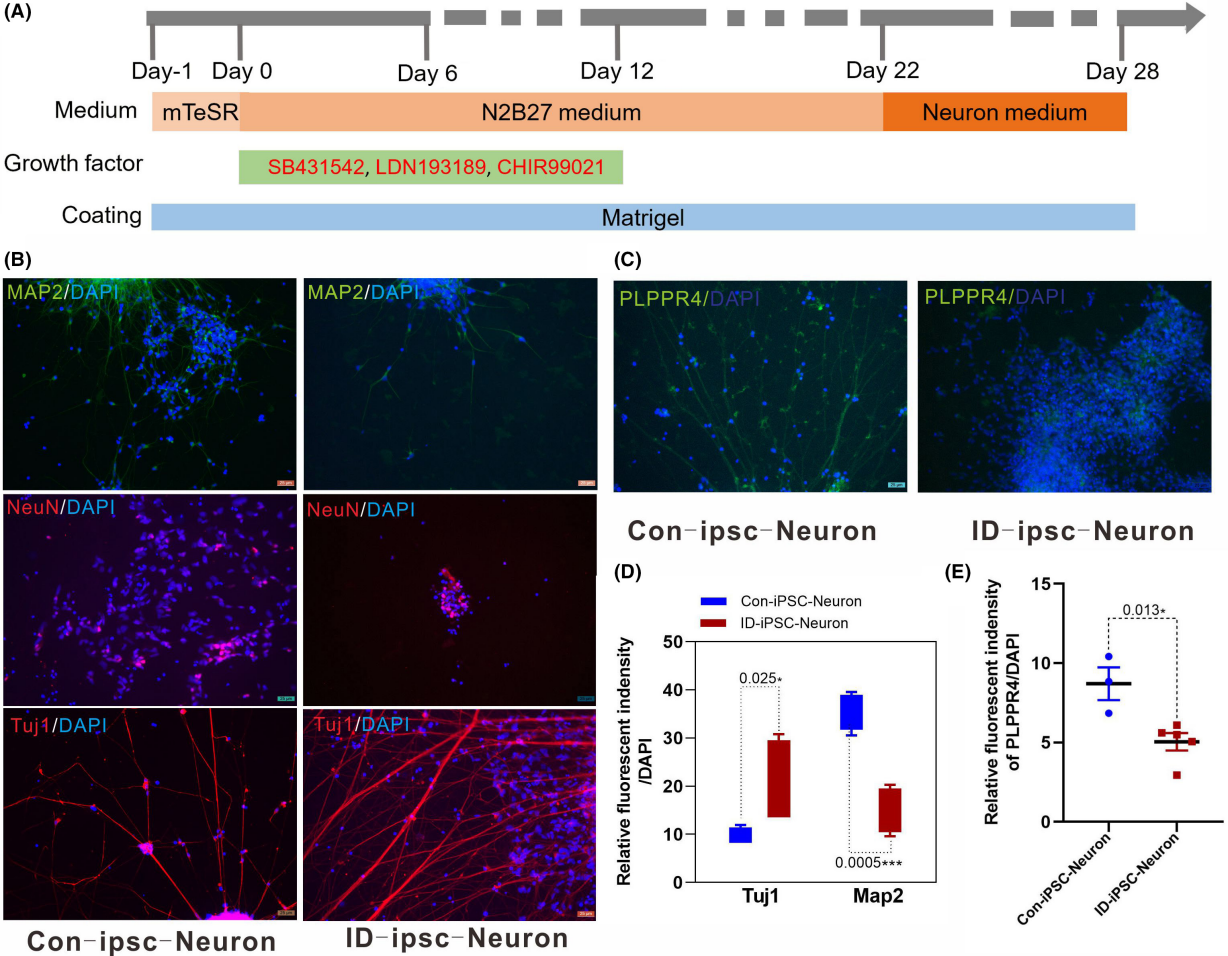
Differentiation and identification of iPSCs. (A) Schematic diagram of the neuron differentiation protocol. (B) Representative images of a neuron expressing the neuronal markers class III beta‐tubulin (Tuj1), microtubule‐associated protein MAP2 and vertebrate neuron‐specific nuclear protein NeuN on Day 28 of terminal differentiation. Scale bar, 25 μm. (C) Representative immunostaining of PLPPR4 in Con‐iPSC‐neurons and ID‐iPSC‐neurons. Nuclei are stained in blue with DAPI. Scale bar, 25 μm. (D) Box‐and‐whisker quantification of Tuj1 staining revealed significantly higher fluorescent intensity in ID‐iPSC‐neurons compared to Con‐iPSC‐neurons and MAP2 staining revealed significantly lower fluorescent intensity in ID‐iPSC‐neurons compared to Con‐iPSC‐neurons. P‐values are indicated, unpaired Student's *t*‐test. (E) Quantification of PLPPR4 staining revealed significantly lower fluorescent intensity in ID‐iPSC‐neurons compared to Con‐iPSC‐neurons, indicating reduced PLPPR4 expression. *p*‐Values are indicated, unpaired Student's *t*‐test.

Here, we apply SMOM, an alternative approach, to detect gene fracture sites, and the fusion sites were verified by Sanger sequencing (Figure 2B,C). SMOM analysis of the patient 1 generated interpretable chromosomes 1, 8 and 10 translocation profiles from the aligned reads and identified the fracture sites allowing precise determination of the genotypes. An inversion occurs at both fusion sites of chromosome 8, and the inversion break is found in the intron of *LOXL2*, located in 8p21.3. *LOXL2* was associated with tumour suppression, cell adhesion and ageing but not with ID or neurodevelopmental abnormalities.^13^, ^17^

Patient 2 was an 11‐year‐old male with ASD diagnosed at age 2. Other clinical features included short status, delayed motor activity and poor weight gain. He had very low muscle bulk and low muscle tone. However, his cognition was normal. He had stuttered during school, with a mixed receptive‐expressive language disorder. His work‐up was unremarkable except for his whole exome sequencing (WES) analysis that showed he has a nonsense mutation in *PLPPR4* (NM_014839, c.4C > T, p.Gln2*).

Patient 3 was an 8‐year‐old Caucasian male with infantile spasms, complex partial seizures, developmental delay and hypotonia. His examination showed relative macrocephaly, a bulbous nasal tip and a flat nasal bridge. He would make only fleeting eye contact and be constantly moving. He was diagnosed with cortical visual impairment. At 8 months, he has admitted for 72 h VEEG monitoring and diagnosed with infantile spasms with modified hypsarrhythmia. He has a variant in *PLPPR4* (NM_014839: c.408 + 2 T > C), and this splice site variant destroys the canonical splice donor site in intron 2. It is predicted to cause abnormal gene splicing, leading to an abnormal message subject to nonsense‐mediated mRNA decay or an abnormal protein product if the message is used for protein translation.

### 3.2 Neuronal differentiation of iPSCs

To recapitulate PLPPR4 pathology in vitro, we generated iPSCs from the PBMCs of patient 1.^14^ The PLPPR4‐iPSCs and control iPSCs were cultured in Matrigel after two generations. Cells were in good condition and not differentiated, considered the starting point of differentiation (Figure 2A). Neural stem cells were directly induced from iPSCs in vitro and then differentiated into neurons.

We relied on immunostaining to identify neurons during neuronal differentiation. To evaluate whether PLPPR4 is involved in the differentiation of iPSC, we used MAP2, NeuN and Tuj1 for immunofluorescence staining at 28 days of differentiation. MAP2 is a neuron‐specific cytoskeleton protein that stabilizes microtubules regulating neuronal polarity and dendritic extension.^18^ Tuj1 is a microtubulin protein that is thought to be involved in neuronal cell type‐specific differentiation.^19^ Neuronal nuclear antigen (NeuN) is a nuclear protein widely expressed in mature postmitotic neurons.^20^ The staining for MAP2, NeuN and Tuj1 clearly shows that all iPSC were positive. It indicated that these cells have neuronal features, which project an extensive network of neurofilaments (Figure 2B). They form branches of more elongated neurites projections connecting surrounding neurons.

It had reported that PLPPR4 is specifically expressed in the brain.^7^, ^8^ Since *PLPPR4* plays a crucial role in synaptic plasticity, we used anti‐PLPPR4 to immunostain late‐stage differentiated neurons. Both types of neurons expressed PLPPR4 (Figure 2C), but the staining revealed significantly lower fluorescent intensity in PLPPR4‐ iPSCs (patient‐derived iPSCs) neurons (Figure 2E), indicating reduced PLPPR4 expression. Then, we used MAP2, NeuN and Tuj1 to examine the development of neuronal axons and dendrites. MAP2 staining revealed significantly lower fluorescent intensity in PLPPR4‐iPSCs neurons. Conversely, levels of Tuj1 were increased significantly compared to control neurons (Figure 2D). MAP2 expressed first in neural precursor cells with subsequent increased expression upon neuronal maturation.^18^ In contrast, Tuj1 was present in immature neuronal cell bodies, dendrites, axons and axon terminals.^19^ However, impaired neural differentiation was predicted by fluorescence staining of MAP2 and Tuj1 in the neuron stage. The data suggest that PLPPR4 may be an essential regulator of human neuronal differentiation and maturation. The PLPPR4 haploinsufficiency can impair neuronal differentiation.

### 3.3 *PLPPR4* plays a critical role in neurite morphology

**FIGURE 3:**
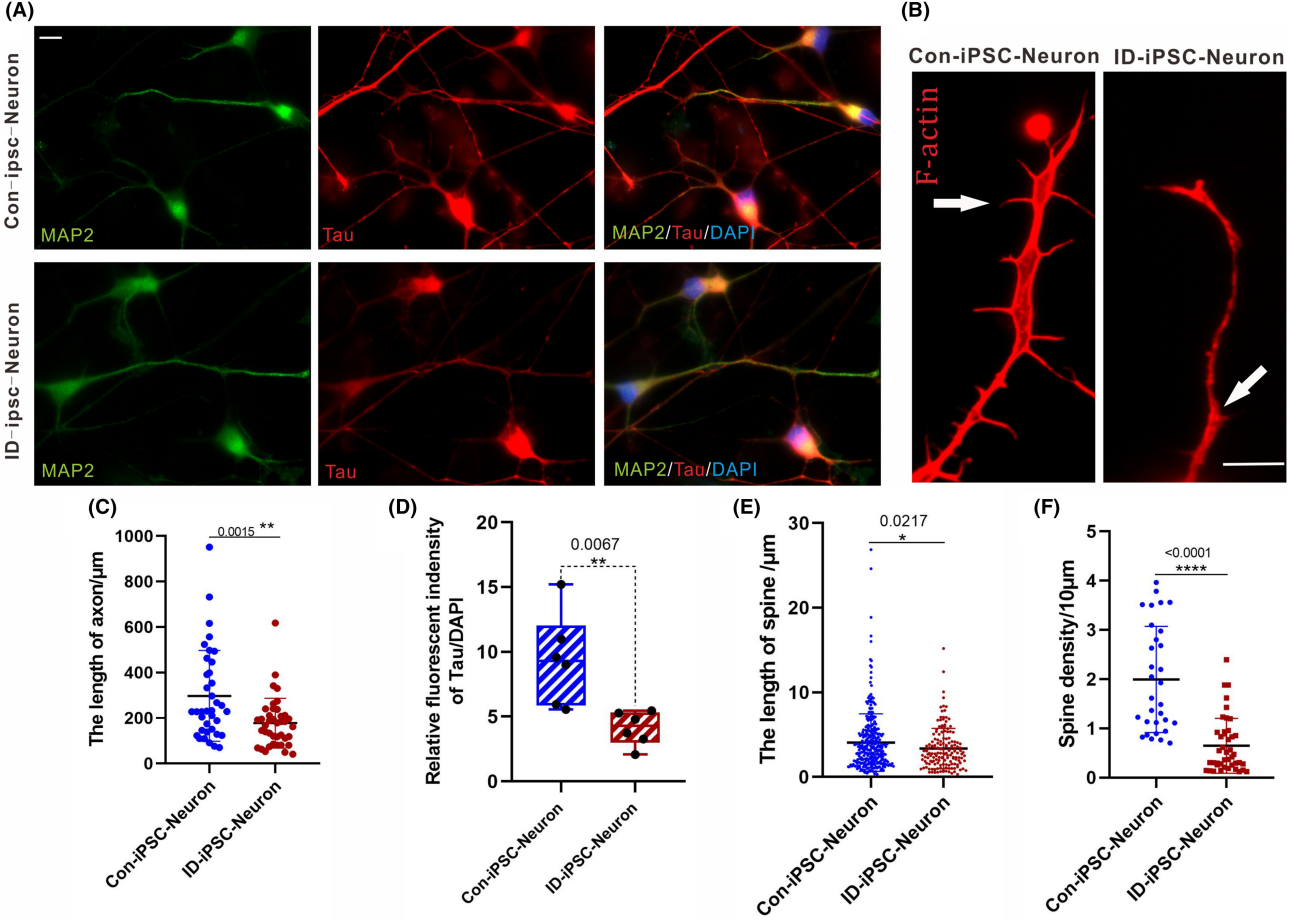
Impaired neurite morphology and ion currents of iPSC‐derived neurons from an ID patient. (A) Representative images of a neuron expressing the neuronal markers MAP2 and Tau. Scale bar, 10 μm. (B) Neurons stained with Phalloidin‐iFluor 594 Reagent‐CytoPainter probe (red fluorescence, F‐Actin). Arrowheads point to dendritic protrusions. Graph representing decreased dendritic protrusions on neurons from an ID patient on Day 28 of terminal differentiation. Scale bar, 10 μm. (C) Quantitative analyses of the neuronal axon length per neuron. *p*‐Values are indicated, unpaired Student's *t*‐test (Con‐iPSC‐neurons = 37, ID‐iPSC‐neurons = 40). (D) Box‐and‐whisker quantification of Tau staining revealed significantly lower fluorescent intensity in ID‐iPSC‐neurons compared to Con‐iPSC‐neurons. (E) Quantification of the length of spines. *p*‐Values are indicated, unpaired Student's *t*‐test (Con‐iPSC‐neurons = 309, ID‐iPSC‐neurons = 156). (F) Quantification of the number of dendritic protrusions. *p*‐Values are indicated, unpaired Student's *t*‐test (Con‐iPSC‐neurons = 31, ID‐iPSC‐neurons = 43).

Abnormal dendritic development originates from neurodevelopmental disorders such as autism and ID. The dendritic spine is a crucial component of synapses and imperative for interneuronal communication. MAP2 and Tau were significantly enriched in mature neurons, and it has been shown to undergo changes during periods of neuronal structural plasticity.^21^ PLPPR4 dysregulation may dramatically affect brain function.^22^ After neural differentiation, most neurons exhibited distinct morphologies with branched processes, which was well visualized by the immunocytochemistry of MAP2 and Tau. The Image J plugin Analyze Skeleton was used to quantify changes. Compared with the controls, we found a significant decrease in the MAP2 and Tau staining in the PLPPR4‐iPSCs neurons (Figure 3A). This allows confirmation that PLPPR4 downregulation in the PLPPR4‐iPSCs neurons perturbs the dendrite branching and axon formation.

Phalloidin (F‐actin) staining of neuronal dendrites showed a variety of dendritic structures, including mature spines, non‐spiny synapses and immature spines.^23^ Changes in the production of F‐actin lead to subsequent changes in spines and additional dendritic structures, thus making Phalloidin an essential tool for investigating somatodendritic integrity.^24^ Then, to further characterize the effect of the PLPPR4 on neurons, we used red fluorescent‐labelled Phalloidin to stain neurons and evaluated the neurite outgrowth. The branch and neuronal lengths were computed by scoring about 50 neurons per sample. We found that F‐actin puncta decreased in all patient neurons (*p*‐value < 0.05). Regarding controlling, the length of branches and neurons was significantly diminished in PLPPR4‐iPSC neurons. It indicated that PLPPR4 haploinsufficiency affects dendritic development and alters neuron morphology.

### 3.4 Loss of function of PLPPR4 leads to aberrant activation of the mTOR/MARK signalling pathway

**FIGURE 4:**
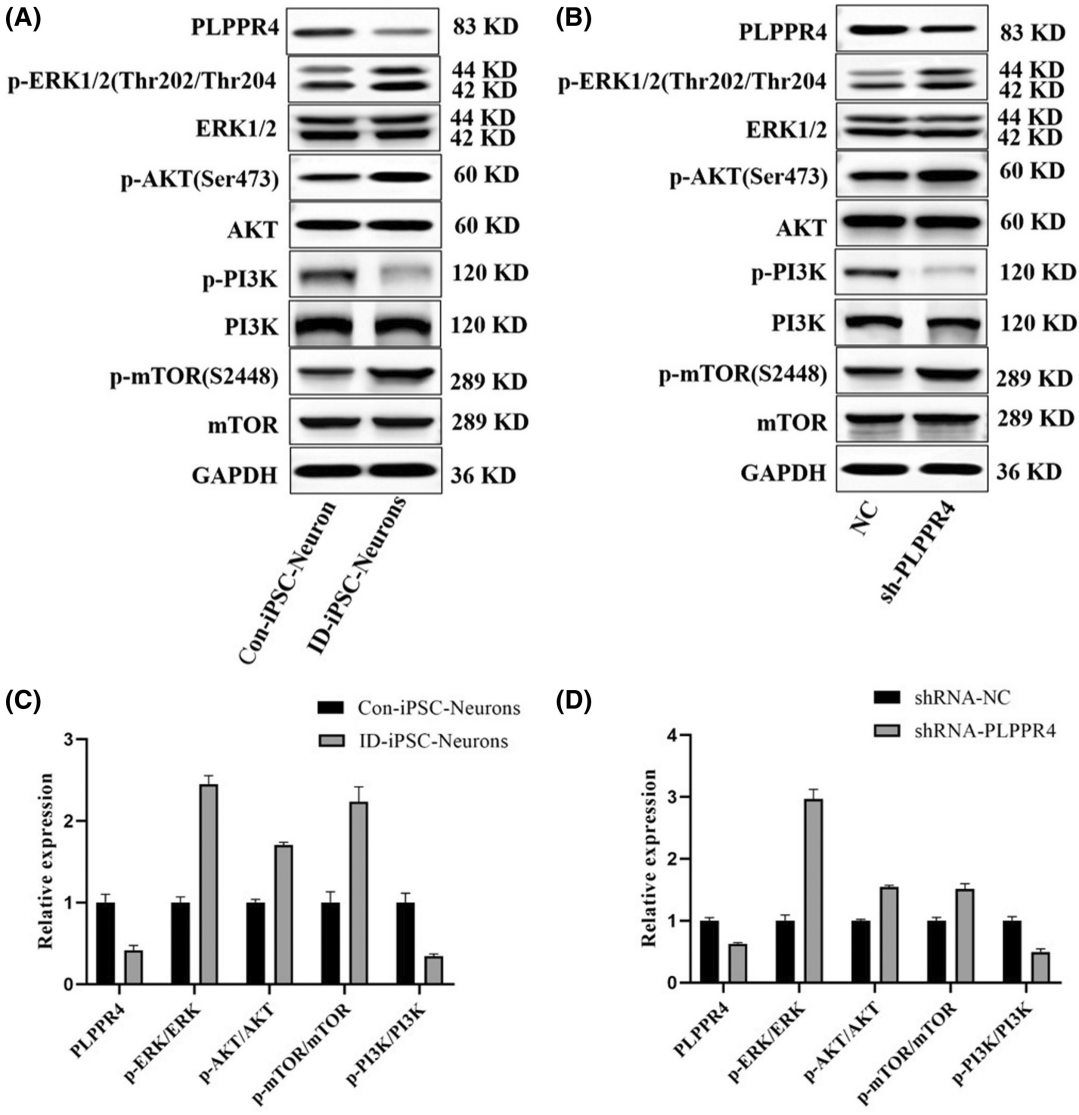
Representative immunoblots of lysates, showing the levels of p‐mTOR, mTOR, p‐AKT, AKT, PI3K, p‐PI3K, p‐ERK1/2, ERK1/2 and PLPPR4 protein levels together with normalizer GAPDH (*n* = 3 biologically independent samples per group). (A and C) Representative immunoblots of lysates from Con‐iPSC‐neurons and ID‐iPSC‐neurons showing phosphorylated and total PI3K (p‐PI3K and PI3K), mTOR (p‐mTOR and mTOR), ERK1/2 (p‐ERK1/2 and ERK1/2) and PLPPR4 levels normalized to GAPDH. (B and D) Representative immunoblots of lysates from a stable shRNA expression system for the *PLPPR4* gene in SH‐SY5Y and control cells, showing the levels of phosphorylated and total PI3K (p‐PI3K and PI3K), AKT (p‐AKT and AKT), mTOR (p‐mTOR and mTOR), ERK1/2 (p‐ERK1/2 and ERK1/2) and PLPPR4 normalized to GAPDH.

Numerous studies have demonstrated that mTOR regulates neuronal differentiation and survival and plays a vital role in the adult brain's synaptic plasticity, learning and memory, and food uptake.^25^, ^26^ mTOR negatively regulates PP2A, and PLPPR4 can regulate synaptic plasticity and brain functional reorganization during neuronal development or after cerebral lesion via PLPPR4/PP2A pathway.^27^ Therefore, using Western blot analysis, we investigated the effect of PLPPR4 on the mTOR signalling pathway in iPSC‐induced neurons. The results revealed that the expression of PLPPR4 was reduced significantly in PLPPR4‐iPSCs neurons compared to the control group. The AKT, p‐AKT, mTOR, p‐mTOR, ERK1/2, p‐ERK1/2, PI3K and p‐PI3K were examined in response to the effect of PLPPR4 in these neurons. The p‐mTOR level is increased in PLPPR4‐iPSCs neurons, hinting that PLPPR4 may inhibit the activation of the mTOR pathway. We also found that the p‐AKT level was significantly upregulated, whereas the expression levels of p‐PI3K decreased significantly in PLPPR4‐iPSCs neurons. These findings support that PLPPR4 haploinsufficiency results in dysregulated mTOR activity throughout neuronal development (Figure 4A).

Interestingly, we also found that PLPPR4 haploinsufficiency can increase the expression of phosphorylation of ERK1/2, suggesting that PLPPR4 may be implicated in regulating the MARK pathway (Figure 4A).

To further confirm the direct involvement of PLPPR4 in the PI3K‐AKT–mTOR and ERK1/2 pathway, shRNA was used to knock down PLPPR4 protein levels in SH‐SY5Y cells. As shown in Figure 4B, PLPPR4 protein levels were successfully reduced in SH‐SY5Y cells. In SH‐SY5Y cells, p‐mTOR, p‐ERK1/2 and p‐AKT were increased, whereas p‐PI3K was reduced. These results strongly suggest that reductions in PLPPR4 lead to downregulating PI3K‐AKT–mTOR activation. It serves as a pathological manifestation of ID or ASD. The study showed that PLPPR4 is a crucial factor leading to mTOR, PI3K and AKT hyperphosphorylation and is reversely regulated by the PI3K‐AKT–mTOR pathway.

## 4 DISCUSSION

In recent years, genetic testing has emerged as a routine clinical tool for the genetic screening of ID and ASD patients. Previously, we reported one case of complex chromosome rearrangement carrying a 0.21‐Mb deletion at 1p21.2p21.3, which included only one coding gene, *PLPPR4*. This patient was diagnosed with mild ID with dysmorphic facial features, hypertelorism and strabismus^4^ (Figure 1A).

Karyotyping, as the principal approach, is routinely performed in the clinic.^28^ However, this inherently low‐resolution and low‐throughput method cannot characterize extensively rearranged genomes. Microarrays are another commonly used method to detect gains and losses of genes, but they do not provide the precise location of rearrangements and cannot detect balanced rearrangements.^29^ The whole genome sequencing technology developed by Bionano, direct labelling and staining (DLS), was used to identify the chromosome‐breaking sites of the patients. The technique can produce an ultra‐long map covering the entire chromosome arm and align the DLS visual map to the physical map of the reference genome transformation to detect potential structural variations.^30^ In this study, we found a balanced translocation between chromosomes 1, 8 and 10 in this patient, and PLPPR4 haploid deletion may occur during the balanced translocation. An inversion occurs at both fusion sites of chromosome 8, and the inversion break is found in the intron of *LOXL2*, which is located in 8p21.3. The LOXL2, a member of the lysyl oxidase (LOX) family, plays a significant role in the cross‐linking and maturation of collagen and elastin, key components of the extracellular matrix (ECM).^31^ Alterations in the ECM can significantly influence a broad range of physiological and pathological processes, including cell adhesion, migration, proliferation, differentiation, as well as tissue morphogenesis, repair and fibrosis. Consequently, the dysregulation of *LOXL2* has been associated with a spectrum of diseases, particularly those connected with ECM remodelling, such as fibrosis and cancer.^32^ Nonetheless, the function of LOXL2 in the central nervous system remains less well‐understood. As of the current state of research, there is no direct evidence linking *LOXL2* to neurological disorders or ID.

We have demonstrated that Bionano SMOM in the hands of an experienced laboratory is an appropriate and straightforward tool to confirm *PLPPR4* fracture sites that contain no suspected pathogenic gene. As such, we speculate this method can provide an ideal complement to genome sequencing for resolving complex genomic architectures. We checked *PLPPR4* in the Exome Aggregation Consortium (ExAC) database, where data showed the gene was highly loss‐of‐function intolerant (pLI = 0.97).

We searched patients with similar clinical phenotypes for *PLPPR4*. Fortunately, two other patients with *PLPPR4* variations were provided by Michael Kruer MD (Phoenix Children's Hospital). One patient had a nonsense mutation in *PLPPR4* (NM_014839, c.4C > T, p.Gln2*), and the other patient had a splice variant in *PLPPR4* (NM_014839: c.408 + 2 T > C). They shared the same clinical phenotype of neurodevelopmental disorder, with mild ID and ASD (Table 1). We speculated that *PLPPR4* might be an associated gene for ID or ASD.

Many studies suggest an involvement of PLPPR4 in neurological diseases such as epilepsy, neurotrauma and memory impairment.^9^ Previous studies have shown that *PLPPR4*‐deficient mice had altered LPA/LPA2‐R signalling.^8^ It can increase the probability of presynaptic glutamate release, which leads to the over‐excitation of neurons and interferes with the development of cortical synaptic connections. It results in epilepsy or hyperkinesia, similar to that observed in mental disorders. The mice with heterozygous deletion of *PLPPR4* had no epileptic performance. Regardless, the excitability of EPSCs was about half that of the mice with homozygous deletion, and PLPPR4 expression was approximately linear with the biological potential.^7^, ^8^ The patient‐specific iPSCs offer an attractive model for understanding disease development. Neuronal development was assessed by investigating neurite morphology and dendritic protrusions, as well as the functional activity of the neurons. The results of this study provide several new insights into the effect of PLPPR4 on neuron differentiation. First, PLPPR4 is highly expressed in neurons. In vitro experiments further demonstrated that PLPPR4 is necessary and sufficient for neuron differentiation. Second, PLPPR4 haploinsufficiency may inhibit neuron maturation. Third, PLPPR4 haploinsufficiency might cause dendrite damage and differentiation with a more prolonged process (Figure 2).

Given that the dendritic spine is a crucial component of chemical synapses and, therefore, imperative for interneuronal communication, in our study, the mean length of protrusions was significantly shorter, and the density of protrusions was markedly lower in the ID patient neurons. These changes uncovered that PLPPR4 might be involved in dendritic spine functions, and *PLPPR4* loss may disturb neurite morphology (Figure 3).

Activation of mTOR is required for the formation, maturation and function of new spine synapses.^33^ Excessive protein synthesis usually results in significant plasticity and behavioural deficits rather than ‘improved’ plasticity. The excess of mTOR signalling in the brain highlights a potential link between mTOR and neurological disease in humans. There is abundant evidence linking mTOR signalling to synaptic change, memory and neurological disease.^34^, ^35^, ^36^, ^37^ As shown in the results, the p‐mTOR and p‐AKT levels were increased. In contrast, the expression levels of p‐PI3K decreased significantly compared to the control neurons, hinting that PLPPR4 may inhibit the activation of the mTOR pathway. These findings indicate that the PLPPR4 haploinsufficiency may impair the mTOR signalling pathway, leading to abnormal synaptic nerve activity.

ERK1/2 is part of a signalling cascade that is activated in response to extracellular signals, such as neurotransmitters or neurotrophins.^38^ It affects synaptic recombination and axon growth.^39^ To clarify the role of ERK1/2 in abnormal axon and dendrites growth, we found that p‐ERK1/2 expression levels were increased in PLPPR4‐iPSCs neurons. Our results suggest that inhibition of PLPPR4 induces the expression of p‐ERK1/2, which is associated with dendritic and synapse growth. It suggested that the PLPPR4 may participate in abnormal axon and dendrites growth by regulating the ERK1/2 signalling pathway. Since abnormal axon and dendrites growth is closely related to neuroplasticity, an important pathological component of neurological diseases, we believe that the ERK1/2 signalling pathway may be essential in the occurrence and development of ID and ASD.

Morphological studies showed that iPSC neurons from ID patients had abnormal differentiation and decreased neuron maturity. Furthermore, the protrusions and neuron lengths were reduced, indicating that the patient had a nerve cell elongation disorder. In summary, these data demonstrate that the mTOR and ERK1/2/MARK pathway is associated with neuroplasticity, so the loss of function of PLPPR4 results in the abnormal activation of the mTOR signalling pathway, leading to disruption of neuronal function. It suggested that PLPPR4 drives a cell‐autonomous signalling pathway, which may regulate dendritic spine morphology, and subsequently impaired cognitive function and potentially other neurodevelopmental disorders, including ID and idiopathic ASD in humans. It is the first time PLPPR4 has been associated with human disease. The findings shed light on the underlying mechanism of neurodevelopmental disorder caused by *PLPPR4* mutations and suggest that *PLPPR4* is a potential target for gene therapy in disorders of neuronal development.

## AUTHOR CONTRIBUTIONS

**Huanzheng Li:** Conceptualization (equal); data curation (equal); resources (equal); writing – original draft (equal); writing – review and editing (lead). **Qian Zhang:** Data curation (equal); writing – original draft (equal). **Ru Wan:** Validation (equal). **Lili Zhou:** Resources (equal). **Xueqin Xu:** Resources (equal). **Chenyang Xu:** Resources (equal). **Yuan Yu:** Methodology (equal). **Yunzhi Xu:** Resources (equal). **Yanbao Xiang:** Resources (equal). **Shaohua Tang:** Conceptualization (equal); data curation (equal); resources (equal).

## CONFLICT OF INTEREST STATEMENT

The authors declare no conflict of interest regarding the publication of this paper.

## Data Availability

The data and materials used and analysed during the current study are available from the corresponding author on reasonable request.
